# Metabolic-Dysfunction-Associated Steatotic Liver Disease (MASLD) after Liver Transplantation: A Narrative Review of an Emerging Issue

**DOI:** 10.3390/jcm13133871

**Published:** 2024-06-30

**Authors:** Alberto Savino, Alessandro Loglio, Flavia Neri, Stefania Camagni, Luisa Pasulo, Maria Grazia Lucà, Roberto Trevisan, Stefano Fagiuoli, Mauro Viganò

**Affiliations:** 1 Gastroenterology Hepatology and Transplantation Unit, ASST Papa Giovanni XXIII, Piazza OMS 1, 24127 Bergamo, Italy; alberto_savino@icloud.com (A.S.); sfagiuoli@asst-pg23.it (S.F.); 2 Gastroenterology, Department of Medicine, University of Milan Bicocca, 20126 Milan, Italy; 3 Department of Organ Failure and Transplantation, ASST Papa Giovanni XXIII, 24127 Bergamo, Italy; 4 Endocrine and Diabetology Unit, ASST Papa Giovanni XXIII, 24127 Bergamo, Italy; 5 Department of Medicine and Surgery, University of Milano Bicocca, 20126 Milan, Italy

**Keywords:** liver transplant, metabolic-dysfunction-associated steatotic liver disease, MASLD, metabolic-associated steatohepatitis, MASH

## Abstract

The development of steatotic liver disease after liver transplant (LT) is widely described, and epidemiological data have revealed an increased incidence in recent times. Its evolution runs from simple steatosis to steatohepatitis and, in a small proportion of patients, to significant fibrosis and cirrhosis. Apparently, post-LT steatotic disease has no impact on the recipient’s overall survival; however, a higher cardiovascular and malignancy burden has been reported. Many donors’ and recipients’ risk factors have been associated with this occurrence, although the recipient-related ones seem of greater impact. Particularly, pre- and post-LT metabolic alterations are strictly associated with steatotic graft disease, sharing common pathophysiologic mechanisms that converge on insulin resistance. Other relevant risk factors include genetic variants, sex, age, baseline liver diseases, and immunosuppressive drugs. Diagnostic evaluation relies on liver biopsy, although non-invasive methods are being increasingly used to detect and monitor both steatosis and fibrosis stages. Management requires a multifaceted approach focusing on lifestyle modifications, the optimization of immunosuppressive therapy, and the management of metabolic complications. This review aims to synthesize the current knowledge of post-LT steatotic liver disease, focusing on the recent definition of metabolic-dysfunction-associated steatotic liver disease (MASLD) and its metabolic and multisystemic concerns.

## 1. Introduction

Excessive fat accumulation in the liver (formally known as steatosis), with a minimum threshold of 5% of the hepatocytes involved, has classically defined the nosological entity of non-alcoholic fatty liver disease (NAFLD) [1]. Since its identification in the late 1980s, the burden of NAFLD has rapidly grown, making it one of the most common chronic liver diseases worldwide, with a current overall prevalence of up to 38% in some regions [2]. The portrait of liver transplant (LT) has been strongly influenced by this and other changes, and NAFLD currently represents the second-leading indication for LT in the United States and one of the first in Europe [2,3,4].

In the past decades, rising evidence has revealed a tight epidemiological and pathogenetic connection between NAFLD and the metabolic dysfunction state, primarily caused by insulin resistance. Cardiometabolic risk factors (CMRFs), such as hyperglycemia, hypertension, abdominal obesity, and dyslipidemia, have been associated with this condition [5]. Parallelly, in subjects with NAFLD and CMRFs, higher mortality has been reported due to both hepatic and extra-hepatic causes, such as cardiovascular (CV) diseases, cancers, and type 2 diabetes mellitus (DM2) [6,7,8]. All these elements have increased the awareness of this condition and have recently led to the proposal of new definitions of metabolic-dysfunction-associated steatotic liver disease (MASLD) and metabolic-dysfunction-associated steatohepatitis (MASH), replacing the old definition of NAFLD and updating the definitions of both the former metabolic-dysfunction-associated fatty liver disease (MAFLD) and non-alcoholic steatohepatitis (NASH).

MASLD is now defined as the presence of hepatic steatosis in conjunction with at least one CMRF and no other discernible causes of steatosis, whereas steatosis with the presence of inflammation (in the lobular or portal side) and hepatocyte injury characterizes MASH [9]. Virtually, patients with the former diagnosis of NAFLD could be almost completely included in these new definitions, as demonstrated by an analysis of the European NAFLD registry and other studies, where more than 98% of patients with NAFLD have met MASLD’s criteria [8,9,10,11].

**Figure 1:**
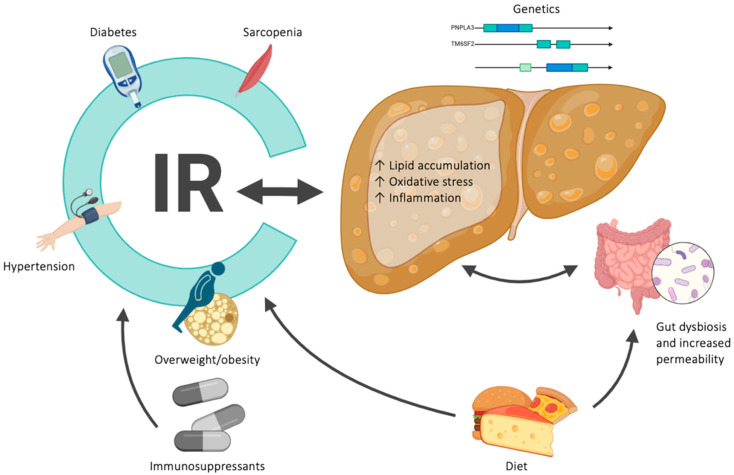
A schematic picture of the factors involved in the pathophysiology of MASLD. Abbreviations: metabolic-dysfunction-associated steatotic liver disease (MASLD); insulin resistance (IR).

The pathogenesis of MASLD is not fully understood, but it is certainly a complex, multifactorial process (Figure 1).

Metabolic syndrome (MS) is strongly linked to liver steatosis, essentially through the mechanism of insulin resistance (IR), in turn resulting from disrupted glucose and lipid metabolism. Chronic hyperglycemia contributes to several pathological processes, including persistent low-grade inflammation and oxidative and endoplasmic reticulum stress (glucotoxicity). In a steatotic graft after LT, hepatic lipid accumulation is driven by four key mechanisms, namely excessive lipid uptake, de novo lipogenesis, β-oxidation of fatty acids, and reduced export of lipids from the liver, each influenced by the synthesis and interaction of pro-inflammatory cytokines, adipokines, and leptin. High dietary intake of carbohydrates (such as glucose, fructose, and sucrose) and lipids is linked to metabolic syndrome and gut dysbiosis. Microbial imbalances and increased intestinal permeability affect liver homeostasis through the gut–liver axis, involving several mechanisms. Primarily, pro-inflammatory microbial metabolites, such as lipopolysaccharides, and disrupted bile acid metabolism, mediated by farnesoid X receptor (FXR), increase hepatic fat accumulation and inflammation. Additional mechanisms include the production of short fatty acids, ethanol, and acetate. Parallelly, immunosuppressive (IS) drugs play a central role in metabolic alteration, widely affecting weight gain, dyslipidemia, arterial hypertension, and glucose homeostasis. In the background, a genetic predisposition, related to definite variant alleles, such as PNPLA3 or TM6SF2, can enhance the process of liver fat storage. Moreover, sarcopenia is correlated with IR and steatosis due to chronic low-grade inflammation, sustained by myokine, adipokine, and miRNA production. All these mechanisms can induce enhanced stress and the damage of hepatocytes, leading to persistent inflammation and fibrosis development [5].

In the post-transplant setting, the presence of steatosis has been widely reported, too, and appears to be common [12]. Formally, it is classified into two different subtypes, recurrent and de novo forms, depending on the etiology of the liver disease that has led to the LT. Recurrent NAFLD/MASLD refers to the re-emergence of steatotic disease in patients who required a LT for NASH-/MASH-related cirrhosis or related hepatocellular carcinoma (HCC), while the occurrence of new-onset steatosis in patients who underwent a liver transplant because of liver disease of other etiologies characterizes de novo NAFLD/MASLD.

The importance of the early recognition and subsequent proper management of this condition lies in the possible development of liver fibrosis and cirrhosis and its contribution to CV disease and malignancy-related mortality. However, given the peculiar setting of LT with multiple variables and confounding factors, such as the use of immunosuppressants, with their metabolic effects, or the nearly constant weight gain, the issue of the generalizability of the new MASLD definition to the post-transplant context has been raised [13].

At present, since the recent proposal of MASLD/MASH criteria, there is a paucity of studies on this specific matter in this setting. In contrast, lots of evidence exists about recurrent or de novo NAFLD and NASH, leading to the use of these two terms as synonyms of MASLD and MASH.

This review provides an overview of the current literature on the burden and features of steatotic liver disease after LT, from the epidemiological burden and natural history to management options, going through risk factors, metabolic and multisystemic involvement, and diagnostic tools.

## 2. Disease Burden: Epidemiology, Natural History, and Socio-Economic Impact

### 2.1. Epidemiology

In the post-LT setting, the presence of steatosis has been largely studied, and available data show a high incidence and prevalence of steatosis, with a time-dependent increase after LT. However, there is wide variability and heterogeneity across studies, and none of them have specifically adopted the recent definition of MASLD, with the former definition of NAFLD being essentially used (Table 1).

Recently, a meta-analysis of 40 studies, mainly retrospective and using liver biopsy for diagnosis, showed a weighted post-LT prevalence of steatosis of around 39% (ranging from 12% to 88%), with wide geographical heterogeneity, as the United States and Latin America showed a higher prevalence compared to Europe and Asia [12]. Quite similar results were reported in a previous large meta-analysis performed by Saeed et al. and also by studies in which the presence of steatosis was specifically assessed by vibration-controlled transient elastography (VCTE) or based on the more recent MAFLD criteria [14,15,16,17]. The same prevalence (33%) was also described in a prospective cohort of patients who received LTs from living donors in India [18]. In line with the growing prevalence of steatotic disease in the general population, an increasing trend through the years has also been described in the post-LT setting, amounting to 11% for each decade [12,19]. The risk of developing recurrent NAFLD seems 5-fold higher than that of developing de novo NAFLD [12,17,20,21].

### 2.2. Natural History

The natural history of fat accumulation in the liver of patients after LT is less studied than in general population, where it seems superimposable between NAFLD and MASLD [10]. As known, the spectrum of MASLD encompasses simple steatosis to steatohepatitis until the development of fibrosis, cirrhosis, and/or HCC. It is associated with high mortality, mainly due to CV disease and metabolic complications, in addition to non-hepatic malignancies and liver failure, which is strongly linked to the fibrosis stage [1,22,23].

The onset of both NAFLD and NASH seems highly variable in relation to LT. Indeed, the former has been reported as early as 3 weeks up to more than 10 years after LT, whereas the latter has been reported from 6 weeks to 15 years after LT [12,17,21,24]. Globally, the occurrence of steatotic graft disease appears to increase with time after LT, and its early occurrence seems associated with a higher risk of developing NASH [24,25]. The rate of progression from simple steatosis to steatohepatitis is estimated to be less than 20%, and its overall occurrence is reported to be 27–29% [12,26]. Similar data were described by Saeed et al. for over 5 years after LT, whereas another meta-analysis, involving 2166 patients assessed with liver biopsy, reported lower (2%) occurrence of de novo NASH, indicating the heterogeneity among studies [17,20]. The prevalence of NASH does not seem to differ because of the geographical location or the publication year, and in line with post-LT NAFLD occurrence, recurrent NASH appears to be more frequent and to occur earlier than de novo NASH [12,17,21,27].

Regarding fibrosis, in NAFLD post-LT recipients, its overall occurrence is reported at around 14%, with a low global risk of advanced fibrosis and/or cirrhosis [12]. Fibrosis seems to be both more frequent and advanced in recurrent disease than in de novo disease, especially when the recurrence is early (<12 months), as well as in a more severe grade of steatosis, in steatohepatitis, and in younger patients [17,18,21,24,28,29]. Again, data are not aligned, as a large retrospective histological-based study failed to show any association between significant steatosis and a higher fibrosis stage, whereas Balitzer et al. described a major incidence of advanced fibrosis in de novo NASH compared to recurrent NASH (42% vs. 10%) [27,30]. The development of fibrosis may occur soon in some patients, even within the first year after LT, as described by Dureja et al., but its prevalence increases in a time-dependent manner [24,31,32,33]. Interestingly, fibrosis progression seems to be faster than in the general population, as Galvin et al. described one stage of fibrosis progression over 2.5 years in the LT setting versus 7 years in the non-LT setting, whereas other studies have reported a high prevalence of advanced fibrosis at just 44 months post-LT [18,34,35,36].

In the setting of LT, many factors, other than the baseline disease and patients’ comorbidities, are implicated in patients’ survival, such as graft condition, surgical complications, metabolic changes, and immunosuppressant side effects [37,38]. Globally, the leading causes of death among patients with NAFLD after LT tend to be cancer, infections, CV disease, and graft dysfunction [29,34]. Somehow, other counterintuitive studies have been consensual in showing no influence of NAFLD on overall survival and graft function up to 15 years after LT compared to control groups [12,28,35,39]. However, conclusions regarding the role of post-LT NASH occurrence have been divergent: Narayanan et al. did not report an impact on graft and patient survival, while Gitto et al. reported higher mortality related to histologically proven de novo NASH [25,35,40]. Although evidence has not highlighted a substantial impact of NAFLD/NASH on post-LT overall survival, a trend of higher morbidity has been reported [35,39]. Particularly, CV events seem to be frequent and more commonly associated with metabolic alterations, recurrent disease, and NASH development [25,40,41,42]. At 3 and 8 years after LT, CV disease occurrence has been reported in up to 15.3% and 30.3% patients, respectively [43]. Ischemic stroke or transitory ischemic attack, peripheral artery disease, myocardial infarction, and angina appear to be the most frequent events throughout the studies. Similarly, a trend of higher occurrence of extrahepatic solid cancer was also reported in steatotic patients compared to non-steatotic patients. Skin (non-melanoma type), urological, head and neck, colorectal, and lung cancers appear to be the most common [39,40].

**Table 1:** Cohort studies assessing post-liver-transplant steatotic liver disease.

Authors (Ref.), Publication Year	Study Design	Type of LT	F-Up *	Patient Nr.	Diagnostic Tools	Prevalence/Incidence	Risk Factors		Other Relevant Results
							Pre-LT	Post-LT	
Adali [14], 2023	Cross-sectional, prospective	LD and DD	NR	122	VCTE + CAP	34%/NR	NR	DM2	-
Mak [16], 2023	Retrospective	LD and DD	NR	549	Biopsy and VCTE + CAP	29%/NR	Cryptogenic cirrhosis	BMI, DM2, AH, dyslipidemia	Advanced fibrosis: 4%
Choudhary [18], 2024	Prospective	LD	62	117	US scan	33%/NR	BMI	Overweight, DM2, dyslipidemia	No effect of HS on OS
Vallin [21], 2014	Prospective	LD and DD	84	91	Biopsy	NR/NR	NR	Recurrent NAFLD more aggressive than de novo NAFLD in terms of NASH and fibrosis development	At 5th year: NASH 17%, severe fibrosis 12%
Villeret [24], 2023	Retrospective	LD and DD	56	150	Biopsy	NR/at 1st and 5th years: NAFLD 68% and 85% NASH 15% and 60%	BMI ≥ 31, ≥65 years	Low HDL cholesterol, CsA	At 5th year: F ≥ 2 and F3/4, 48% and 20%
Narayanan [25], 2019	Retrospective	NR	120	588	Biopsy and US scan, CT, MR	NR/at 10th year: 48% (85% de novo vs. 15% recurrent)	Male sex, HCV, NASH	BMI	No effect of HS on survival; NASH is a risk factor for CV events
Miyaaki [26], 2019	Retrospective	LD	48	100	Biopsy	NR/33%	Younger age	Donor steatosis, weight gain	NASH 27%
Balitzer [27], 2022	Retrospective	NR	NR	56	Biopsy	NR/de novo and recurrent NASH: 13% vs. 6.5%	NR	NR	F3/4 in de novo vs. recurrent NASH: 10% vs. 42% but no difference in OS
Yalamanchili [29], 2010	Retrospective	NR	NR	257	Biopsy	NR/At 1st, 2nd, 5th, and 10th years: 8%, 14%, 25%, and 33%	NR	NR	At 5th and 10th years: F4 5% and 10%, respectively
Hejlova [30], 2016	Retrospective	NR	65	548	Biopsy	NR/At 1st and 10th years: 30% and 48%, respectively	ALD, female, BMI	BMI, triglycerides, alcohol consumption, DM2	NASH: 10%; HS not associated with either higher fibrosis stage or survival
Dureja [32], 2011	Retrospective	NR	82	88	Biopsy	NR/9%	BMI	BMI, triglycerides, prednisone dose at 6th months after LT	F3/4: 3.4%; no effect of HS on OS
Malik [33], 2009	Case–control, retrospective	LD and DD	36	98	Biopsy	NR/70%	Younger age	Younger donor age and BMI, DM2, dyslipidemia, and MS	Recurrent NASH: 25%, F ≥ 2: 18%; no effect of HS on OS
Bhati [34], 2017	Retrospective	NR	NR	103	Biopsy and VCTE + CAP	NR/88%	Female	Glucose and triglyceride levels	F 3/4: 27%; NASH: 41%; cirrhosis: 5% No OS difference among recurrent NAFLD vs. NASH
Galvin [35], 2019	Retrospective	NR	36	430	Biopsy	NR/33%	HCV	DM2, BMI, weight gain, and SIR	F ≥ 2: 40%; no effect of HS on OS
Tejedor-Tejada [39], 2021	Cross-sectional, retrospective	NR	60	252	NITs	36%/NR	Male	Obesity, MS, DM2	F ≥ 2: 58–86%;no effect of HS on OS
Gitto [40], 2018	Retrospective	LD and DD	120	194	Biopsy and NITs	NR/20%	NR	MS, DM2, LD, Tac	Significantly lower long-term OS in patients with de novo NASH

* Median in months. Abbreviations: vibration-controlled transient elastography (VCTE); arterial hypertension (AH); non-invasive tests (NITs); not reported (NR); living donor (LD); deceased donor (DD); hepatic steatosis (HS); cyclosporin (CsA); tacrolimus (Tac); sirolimus (SIR); ultrasound scan (US); computer tomography (CT); magnetic resonance (MR); metabolic syndrome (MS); alcoholic liver disease (ALD); body mass index (BMI); diabetes mellitus 2 (DM2); non-alcoholic steatohepatitis (NASH); hepatitis C virus (HCV); non-alcoholic fatty liver disease (NAFLD); liver transplant (LT); overall survival (OS); controlled attenuation parameter (CAP); high-density lipoprotein (HDL); year (yr).

### 2.3. Socio-Economic Impact

From a socio-economic perspective, LT is a complex, multidisciplinary procedure that consumes a significant amount of resources [44,45]. In the general population, a high socio-economic burden of NAFLD and NASH has also been described, in terms of the cost of care, social impact, and work productivity, and this is especially true for more advanced stages of the disease and for NASH itself. Curiously, the global burden seems more influenced by indirect costs than direct medical costs [46,47,48,49,50]. Unfortunately, no studies have assessed the additional burden of NAFLD/MASLD in the post-LT setting. However, it is reasonable to speculate that it may be substantial, given the high prevalence, the intrinsic connection with metabolic alterations, and the related morbidity. Following the growing trend of incidence and prevalence in the past decades, this burden will likely continue to rise. There is certainly an unmet need for data focusing on this concern to quantify both direct and indirect costs better, as suggested by a recent panel of experts, too [51].

## 3. Risk Factors

The risk factors implicated in the development of post-LT steatosis and steatohepatitis can be divided into recipient and donor factors and, depending on the onset timing, pre- and post-LT factors [5,52].

Donor-related factors do not apparently have an impact on post-LT steatosis. Although without statistical significance, a mild association has been reported between graft steatosis and increased donor body mass index (BMI) [12]. The development of steatohepatitis has also been associated with a younger age of the donor, a living donor’s graft, graft steatosis, and the donor’s BMI [12,26]. Globally, these results may suggest the liver’s predisposition to fat accumulation and disease progression, likely due to specific genetic phenotypes.

In line with this hypothesis, some genetic factors have been highlighted among studies, in both donor and recipient. The patatin-like phospholipase domain-containing 3 (PNPLA3) *rs738409-G* variant, also known as PNPLA3 I148M polymorphism, was the first and firmly recognized risk factor for steatosis [53,54]. In both donor and recipient, this allele, either in heterozygosity or in homozygosity, has been described as related to post-LT steatosis in a dose-dependent fashion. Indeed, when present in both donor and recipient, it synergically increases the risk (up to 29-fold) of developing early steatosis [12,55,56,57]. In addition, its effect is amplified by concurrent recipient overweight or obesity [58]. The transmembrane 6 superfamily member 2 (TM6SF2) *rs58542926-A* allele has been proposed as another genetic risk factor associated with more severe steatosis, necro-inflammation, and advanced fibrosis. Its predisposition seems to be additive with PNPLA3 I148M polymorphism and influenced by the recipient’s adiposity, too [59]. Notably, Liu et al. did not find a significant relationship with this variant in their study with a long follow-up of 10 years [58]. The recipient’s superoxide dismutase-2 (SOD-2) *rs4880-A* variant and the donor’s HSD17B13 *rs6834314* variant have been associated with a reduced risk of NAFLD occurrence [60]. Also, many other single-nucleotide polymorphisms have been identified as potential risk factors for steatosis development, along with those related to adiponectin and vitamin D pathways, among others [60,61,62,63].

Regarding recipient factors, they can be separated into three main categories: baseline factors (such as background disease, age at transplantation, and gender), components of metabolic syndrome (MS), and immunosuppressive drugs.

The baseline disease for which patients have been transplanted plays an important role. Indeed, patients transplanted for NAFLD-associated cirrhosis present more than a 5-fold increased risk of developing steatosis and a 29-fold increased risk for steatohepatitis [12]. Underlying alcoholic liver disease is another widely recognized risk factor, even in the absence or with a limited amount of alcohol intake after LT [12,64,65]. Notably, the combination of both pre-LT alcoholic and metabolic liver diseases may have an additive effect, as described by Vanlerberghe et al. [65]. Also, chronic hepatitis C has been associated with a higher risk of post-LT steatosis, probably due to its recurrence. However, with the spread of new antiviral therapies, its role is bound to become progressively marginal [12].

A younger age at the time of LT has been found to be associated with a higher risk of post-LT steatosis and the female sex with an increased risk of steatohepatitis, suggesting a possibly more aggressive form in young, female patients [12,30,33]. Also, sarcopenia may play a role in post-LT NAFLD occurrence, as suggested by Hong et al. [66].

Particular attention should be paid to metabolic factors. Body weight, arterial hypertension, dyslipidemia, and impaired glucose homeostasis up to established DM2 have been widely reported in NAFLD cohorts [12,16,18,26,34,40,41,67]. They share common pathogenetic roots that converge toward IR, and the LT itself carries a major risk of developing metabolic changes mainly due to the almost constant post-LT weight gain and the metabolic effects of immunosuppressants [20,41,68,69]. Thus, the post-LT steatosis risk seems associated with both pre- and post-LT metabolic risk factors. A strong relationship has been particularly described with pre-LT obesity, DM2, arterial hypertension, post-LT weight gain, obesity (particularly abdominal obesity), MS, and its single components [12,14,60]. Among all metabolic risk factors, a post-LT high BMI (≥30 kg/m^2^) and weight gain (>5 kg) appear to be the most impacting factors [16,17,18,27,42,60,70].

Another important topic is regarding immunosuppressive therapies, generally divided into induction and maintenance schemes. In the former, steroids represent the base, in association with biological immunosuppressive agents, i.e., basiliximab, anti-thymocyte globulin, and alemtuzumab. In the latter, the most used medications are calcineurin inhibitors (CNIs), with tacrolimus (Tac) preferred over cyclosporine (CsA). Antiproliferative agents, such as mycophenolic acid (MPA) and azathioprine (AZA), and mammalian targets of rapamycin inhibitors (mTORis), such as everolimus (EVR) and sirolimus (SIR), are usually used for lowering the toxicity of CNI therapy or in other high-risk settings [71]. As widely known, immunosuppressive drugs induce several metabolic effects, with hyperlipidemia, arterial hypertension, weight gain, and DM2 being the most frequent for CNIs or steroids and hyperlipidemia for mTORis (Table 2) [71]. Among all immunosuppressive agents, only antiproliferative agents, such as MPA and AZA, have neutral effects on metabolism and have not been associated with significant metabolic alterations. Surprisingly, no significative relationship between immunosuppressive therapy and steatosis occurrence or fibrosis progression, even for steroids with their known steatogenic effects, has been reported so far [14,17,34,41,43]. To note, only one meta-analysis found a 68% increased risk of steatosis occurrence with the use of SIR, but as specified by the authors themselves, this may hide an underlying selection bias [12]. Finally, it is worth noting that some metabolic and immune-system-related differences may exist in old, transplanted patients (>65 years old) compared with younger patients, due to aging. Particularly, immunosenescence has been described, as well as changes in drug metabolism and pharmacokinetics. Thus, in this subgroup, the role of immunosuppressive agents might be slightly different, as reported in a recent paper [72].

## 4. Diagnostic Assessment

Compared to NAFLD diagnosis, one of the main changes in the new MASLD criteria is the affirmative rather than exclusionary connotation, with a more inclusive approach, where the exclusion of other hepatic diseases is still not required [9]. Anyway, an accurate assessment of hepatic steatosis and its progression remains pivotal for a correct diagnosis.

Histological evaluation is the gold standard for diagnosing liver steatosis and the only tool for the reliable identification of steatohepatitis. It is also the reference standard for fibrosis assessment and grading [1,22]. Liver biopsy is a safe procedure in the LT setting as well, but it remains an invasive technique, with concerns about the patient’s acceptability and, although rare, possible minor and major side effects, such as pain, bleeding, infections, damage of nearby organs (e.g., pneumothorax), and death in exceptional cases [73,74,75,76]. The main drawbacks of this procedure are the subjectivity of histological interpretation, with significant intra- and inter-observer disparity, as well as sampling variability due to a small sample size and heterogeneity in fat and fibrosis disposition throughout the liver [77,78].

In the past decades, non-invasive tests (NITs) have been developed to detect and quantify liver steatosis and fibrosis to overcome the disadvantages associated with liver biopsy, with NITs becoming progressively more accessible. They are represented by liver ultrasound (US), VCTE with controlled attenuation parameter (CAP), magnetic resonance (MR), and computed tomography (CT) techniques, alongside multiple serum markers’ scores. In the LT field, little evidence exists about the role of these NITs, even more so in the field of post-LT MASLD.

Abdominal US is an easy-to-use and widespread tool in clinical practice. It has well-known advantages, such as repeatability, non-invasiveness, and low cost, but at the same time, it is operator dependent, with great subjectivity in imaging interpretation. Bidimensional US presents good diagnostic accuracy for diagnosing moderate–severe steatosis [79]. However, it provides only a semi-quantitative assessment, and its sensitivity becomes weak in obese patients and in lesser degrees of steatosis, when fat accumulation involves less than 30% of the whole parenchyma [80,81,82,83]. To overcome these limitations, computer-assisted quantitative US techniques have been developed, using dedicated post-processing software. The most robust parameter with good performance is the hepatorenal index (the ratio between the brightness within a region of interest in the liver and the right kidney) [84]. For fibrosis, bi-dimensional US has low accuracy as it does not provide reliable information unless signs of advanced fibrosis or cirrhosis are already present [80,81]. In this sense, shear wave elastography (SWE), a specific measure based on acoustic radiation force impulse (ARFI), is a more standardized method to quantify liver stiffness, and as suggested by recent guidelines, it may be used to assess liver fibrosis in NAFLD patients and an LT setting [85,86,87,88].

Based on US technology and sharing with it the same advantages but without the operator dependency of classic US examination, VCTE was developed to measure liver stiffness. CAP, a parameter derived from the attenuation of the US by hepatic fat, has been rapidly introduced next to VCTE for steatosis assessment, providing a semiquantitative evaluation [85,86,88,89]. It appears reliable as a screening tool; however, it has poor capability in differentiating the various steatosis grades, and some concerns have emerged in NAFLD patients with a high BMI, where its performance seems lower independently from the probes used [22,90,91,92]. In the LT setting, few studies have used CAP with good overall performance, and since a unique, defined cutoff still does not exist in this context, the threshold of 270–290 dB/m proposed by some authors appears to be reasonably accurate; however, this needs to be validated [14,16,34,93,94,95]. Regarding liver stiffness, VCTE has demonstrated excellent correlation with fibrosis grades in NAFLD patients [88,96,97]. However, in LT cohorts, data are lacking, and only a few studies have been published [98]. Particularly, the meta-analysis by Bhat et al. describes good performance of VCTE in detecting significant liver fibrosis after LT (AUROC ranging from 0.75 to 0.96), and interestingly, the cutoff of 10.5 kPa for significant fibrosis proposed by Siddiqui et al. demonstrates acceptable accuracy [95,99].

Regarding non-US NITs in pre-LT NAFLD patients, MR-based techniques are the most sensitive and specific NITs to detect, grade, and follow up liver steatosis and fibrosis [36,88,100,101,102,103,104]. Regarding steatosis, two of the most effective methods are magnetic resonance spectroscopy (MRS), which identifies spectral peaks corresponding to the protons in triglycerides, and proton density fat fraction (PDFF) measurement, which is the proportion of MR-visible fat’s protons to the sum of MR visible fat’s and water’s protons [105,106]. To the best of our knowledge, after LT, just two studies with these MR techniques have been performed, with promising results. Burian et al. concluded that MRS can distinguish NAFLD, NASH, and non-steatotic LT recipients, and Hájek et al. reported high specificity (73 to 97%) and sensitivity (73 to 100%) of MRS in the identification of mild and moderate–severe steatosis compared to liver biopsy [107,108]. Available data using MR elastography (MRE) for fibrosis evaluation are scarce in the LT field, and only a small meta-analysis of six studies reported good performance in this setting, with AUROC ranging from 0.69 to 0.96 throughout all fibrosis stages, including cirrhosis [109]. Although MR-based techniques are accurate and capable of assessing both hepatic steatosis and fibrosis, they are not widely available and feasible in daily clinical practice and for all patients, being expensive and time-consuming. Consequently, their usage and diffusion are currently limited and often restricted to clinical research.

Non-contrast-enhanced CT may be used to diagnose hepatic steatosis with the liver attenuation index (LAI), a parameter defined as the differences between mean hepatic and splenic attenuation. LAI has been reported to be accurate in diagnosing liver steatosis but only in advanced stages (>30%), and not mild steatosis, due to the low sensitivity [110,111]. Globally, CT has limited use, being less accurate than US- and MR-based techniques and exposing patients to radiation [80]. No studies have assessed CT performances after LT, to the best of our knowledge.

In the past decades, there has been an increased interest in serum biomarkers. Numerous patented and non-patented scores have been proposed and validated to detect the presence of steatosis and fibrosis. They are based on easily accessible clinical and biochemical elements, and in the general population, their use to rule out significant fibrosis is suggested by international guidelines (even though the ones for steatosis have proved to be less accurate) [1,88]. Generally, for steatosis assessments, the main scores are represented by SteatoTestTM (BioPredictive, Paris, France), the fatty liver index (FLI), the hepatic steatosis index (HSI), the lipid accumulation product (LAP), the index of NASH (ION), and the NAFLD liver fat score (NAFLD-LFS) [112,113,114,115,116,117]. Although they have all been validated in pre-LT settings with similar moderate performances, these scores contribute little to the information already gleaned from routine clinical, laboratory, and imaging examinations, proving, therefore, an additional marginal role [88,118]. Similarly, several scores have been developed aiming to predict clinically significant liver fibrosis: the ELFTM (Siemens Healthcare, Erlangen, Germany), FibroMeterTM (Echosens, Paris, France), FibroTestTM (BioPredictive, Paris, France), the ALT/platelet ratio index (APRI), the fibrosis-4 index (FIB-4), and the NAFLD fibrosis score (NFS). The most validated ones are the NFS and the FIB-4. The former is based on six variables (age, BMI, AST/ALT ratio, platelet count, hyperglycemia, and albumin), while the latter is based on four (age, serum aminotransferase levels, and platelet count). NFS and FIB-4 scores have similar high performance in NAFLD patients, with AUROCs of around 0.74–0.80 [119,120]. None of these scores have been validated in the post-LT setting, and few studies have tested the APRI and the FIB-4, with controversial results, but not in the specific field of post-LT MASLD [121,122,123,124,125].

## 5. Management: Preventing Strategies and Treatment Options

The management of MASLD in the context of post-LT is particularly complex due to the interplay of many factors, such as immunosuppressants, graft function, metabolic alterations, and comorbidities, and requires an integrated, multidisciplinary approach. Generally, management strategies can be divided into preventive measures and therapeutic interventions. The former aim to avert the onset of MASLD and may start before LT if risk factors are already present, while the latter focus on managing steatosis or steatohepatitis, ideally preventing further progression of fibrosis to decompensated liver disease, HCC, and/or associated comorbidities, such as CV and renal diseases [126].

MASLD prevention first relies on the early identification and management of known risk factors: DM2, arterial hypertension, hyperlipidemia, and weight gain. Consequently, regular follow-up remains the prerequisite for correct and effective management, as well as prompt interventions once risk factors are detected. Additional attention to malignancy screening should be warranted as increased neoplastic risk after LT exists [28,39,40,127].

Lifestyle modifications should be the backbone of all strategies, both preventive and therapeutical, focused on weight and metabolic risk factor control. They consist of diet modification (i.e., Mediterranean diet and avoidance of alcohol intake and saturated fatty acids) and regular aerobic physical activities. Appropriate nutritional counseling and supervised physical activity are beneficial and should be recommended [28,128,129,130,131].

Since overweight and obesity are probably among the most impactful aspects for the occurrence of both graft steatosis and metabolic alterations (and significant improvements in steatosis/steatohepatitis are achieved even with modest weight loss), other strategies, such as drug modulation and bariatric interventions, could be taken into consideration on a case-by-case setting, although data are scarce in these fields [132,133]. Indeed, in the post-LT setting, orlistat is the only tested drug, showing a good safety profile but, so far, only discrete results [134]. In addition, bariatric surgery has been assessed, with promising long-term results, though concerns about safety and the correct timing of the LT (pre, post, or concomitant) remain [135,136,137,138,139,140]. A valid, less invasive alternative may be represented by bariatric endoscopy, which includes gastric and duodenal devices and techniques such as intragastric balloons and endoscopic sleeve gastroplasty, but once again, data in the LT context are still lacking [141].

Pharmacotherapy is essential to manage the other metabolic risk factors associated with LT and MASLD. As a general rule, drug-to-drug interaction and renal dysfunction must always be assessed [28].

To reduce the risk of major adverse CV events, the target blood pressure for liver transplant recipients should be below 130/80 mmHg, and 125/75 mmHg if renal impairment is present [142]. The choice of an antihypertensive regimen depends on the patient’s profile and comorbidities as most of the main classes are safe and widely used in LT settings. First-line therapy is generally based on calcium channel blockers or beta-blockers, since their vasodilator mechanism is in direct contrast to the renal vasoconstriction of CNIs [143,144,145]. Other molecules, such as angiotensin-converting enzyme inhibitors, angiotensin receptor blockers, and diuretics, have multiple potential benefits, including proteinuria and afterload reduction. Moreover, they may be beneficial in concomitant kidney impairment, DM2, and heart failure [28]. Importantly, obstructive sleep apnea should be actively investigated as it is strongly related to both obesity and hypertension [28].

The European Society of Cardiology recommends restrictive goals for the management of dyslipidemia after solid-organ transplantation (SOT)—i.e., kidney, liver, or heart—with the same strict targets fixed for patients at high and very high CV risk [146]. About hypercholesterolemia, statins and ezetimibe are valid, widely used, and safe options, while hypertriglyceridemia could be efficiently managed with omega-3 fatty acids and fibric acid derivates [147,148,149]. Notably, lipophilic statins are metabolized by cytochrome P450 and may interfere with CNI levels; consequently, they should be administered starting with a low initial dose, followed by progressive titration and monitoring [148]. Similarly, although well tolerated, fibrates should be monitored because of the potential risk of myopathy and kidney impairment [146].

After LT, the standard of care in glycemic control is represented by a stepwise approach consisting of lifestyle modifications, oral antidiabetic medications, and, finally, insulin schemes [150]. Although hypoglycemic agents have not been specifically assessed in the LT context, metformin, rosiglitazone, pioglitazone, and sulfonylureas have been established as safe following SOT, either alone or in association with insulin [151,152,153]. Globally, metformin may be considered the first choice, given its beneficial effect on CV events [154]. Other drug classes could be safe as well in this setting, such as dipeptidyl peptidase-4 inhibitors and glucagon-like peptide 1 receptor agonists (GLP1RAs) [148]. The only class that should be averted is the sodium-glucose co-transporter-2 (SGLT-2) inhibitors, which are related to volume depletion and a higher occurrence of genitourinary infections [71].

GLP1RAs are of particular interest in the MASLD/MASH field, and several GLP1RAs, also in combination with glucose-dependent insulinotropic polypeptide (GIP) agonists, are in various phases of clinical development. Intestinal GLP1 is an endogenous satiation signal whose eating effects are primarily mediated by vagal afferents. GLP1RAs increase pancreatic insulin secretion, decrease glucagon in a glucose-dependent manner, and delay gastric emptying, which suppresses postprandial hyperglycemia and appetite, resulting in reductions in energy intake and body weight. Moreover, increases in insulin secretion and decreases in glucagon secretion reduce hormone-sensitive lipase activity, reducing the hydrolysis of triglycerides and fatty acids released from adipose tissue, which, in turn, reduces fatty acid entry to the liver [155,156,157]. It has been already reported that GLP1RAs decrease glucose levels and body weight and improve CV outcomes in people with DM2. In addition, GLP1RAs have been reported to reduce liver fat content and liver enzymes, lowering oxidative stress and improving hepatic de novo lipogenesis and the histopathology of MASH [158,159,160,161,162]. Recently, in a phase 2 dose-finding, placebo-controlled trial involving patients with biopsy-confirmed MASH and moderate or severe fibrosis, Loomba et al. reported that 52 weeks of once-weekly subcutaneous tirzepatide (GLP1RA and GIP agonist) is more effective than a placebo for the resolution of MASH. The researchers found that the percentage of participants who met the criteria for the resolution of MASH without worsening of fibrosis was 10% in the placebo group and 44%, 56%, and 62% in the 5, 10, and 15 mg tirzepatide groups, respectively. Similarly, the percentage of patients who had an improvement of at least one fibrosis stage without worsening of MASH was 30% in the placebo group and 55%, 51%, and 51% in the 5, 10, and 15 mg tirzepatide groups, respectively [163]. However, despite these interesting results, up to now, only one retrospective study has evaluated the use of GLP1RAs in patients who underwent SOT and had DM2 either pre- or post-transplant. After a mean follow-up of 12 months, the drug was effective in such recipients for glycemic control and weight loss, as well as in the non-transplant population with DM2, without affecting Tac levels, renal function, or transplant outcomes [164].

The role of immunosuppression in LT is crucial, and the metabolic effect of these medications has been largely reported, although its effective influence in steatosis genesis after LT is less clear [12,17,71,165,166]. Tailoring immunosuppression to mitigate metabolic side effects is a potential, feasible strategy [28]. Generally, immunosuppressive protocols should include short-term and low-dose steroids and CNI minimization as steroids are doubtless one of the most steatogenic immunosuppressive drugs and together with CNIs are implicated in the worsening of the metabolic profile [68,167,168]. Furthermore, each immunosuppressor class presents peculiar features that should be considered case by case. For example, MPA has a global neutral metabolic impact, mTORis have a better profile concerning weight control and hypertension compared to CNIs, but conversely, they present a worse impact on serum lipid levels [169]. Moreover, intraclass differences should be considered, i.e., Tac and CsA, where Tac has shown a minor influence on hypercholesterolemia and arterial hypertension, with CsA affecting glycemic homeostasis less [170,171].

Concerning the use of specific drugs for MASLD, no pharmacological therapies are approved either for advanced liver disease or for LT recipients. Only resmetiron—a thyroid hormone receptor-β agonist—was recently approved for adults with moderate-to advanced-liver fibrosis (non-cirrhotic) MASH. To date, vitamin E and pioglitazone (a peroxisome-proliferator-activated receptor—PPAR—γ agonist) have shown some effects in NASH patients, but their safety and efficacy must be assessed in the LT setting [172]. Similarly, saroglitazar, a dual PPAR α/γ agonist, has shown a beneficial influence, not only in the treatment of NAFLD, but also in dyslipidemia and glycemic control in LT recipients [173].

As studies in the field are rapidly expanding, many new drugs may be approved in the coming years. The most researched and promising drugs include PPAR agonists, thyromimetics, fibroblast growth factor 21 (FGF-21) analogs, and modulators of the bile acid pathway.

PPARs are a family of nuclear receptors (α, β/δ, and γ) that regulate several metabolic processes, including lipid and glucose homeostasis, and have shown beneficial effects on hepatic inflammation. Thyromimetics, particularly β receptor agonists primarily expressed in the liver, act on multiple pathways involving lipid metabolism and interfere with fibrosis deposition. FGF-21 is an inducible hormone involved in the regulation of the energy balance, glucose levels, and lipid homeostasis, showing pleiotropic positive effects in the adipose tissue and the liver. Finally, bile acids regulate liver and metabolic homeostasis through nuclear hormone receptors, like the farnesoid X receptor (FXR). FXR activation improves glucose and lipid metabolism and exhibits both anti-inflammatory and antifibrotic actions [174,175].

## 6. Discussion

LT still represents the only therapeutical option for end-stage liver disease. A significant improvement in both patient and graft survival has been observed over time, and nowadays, most patients are still alive 15 years after transplantation, with a median survival of up to 20 years in many cases [38,176,177,178]. This longer timing implies the inevitable exposure of the graft to the potential development of chronic diseases, such as MASLD.

Due to the recent proposal of new diagnostic criteria, the MASLD incidence and prevalence among LT recipients have not been specifically assessed. Using the former NAFLD definition, the occurrence of steatotic disease after LT has been described as consistent, with a significant rate of patients presenting progressive disease with steatohepatitis and advanced fibrosis [12,17,20]. However, the great uncertainty regarding the real occurrence of steatotic disease after LT must be highlighted, mainly due to the high heterogeneity among studies.

Although some authors have hypothesized that NAFLD and MASLD criteria may be almost superimposable in the general population, the same might not hold in the LT context for several reasons.

First, the LT context is peculiar, and the generalizability of the MASLD criteria to this field has been questioned [13]. Indeed, most LT recipients fulfill the MASLD definition because of weight gain (almost universal in this setting). To minimize the risk of overdiagnosis, other features of MS, apart from the BMI and waist circumference, were proposed by Choudhary et al. as additional diagnostic elements [13]. Another aspect possibly influencing the overlap between NAFLD and MASLD criteria concerns the identification of related risk factors. Particularly, pre- and post-LT metabolic alterations, such as hyperlipidemia, arterial hypertension, DM2, and overweight/obesity, are the main predictive factors of NAFLD/NASH occurrence. However, in the MASLD context, these metabolic alterations cannot be considered risk factors, since they are key elements of the disease itself.

Second, post-LT NAFLD has not been clearly correlated with lower survival or high morbidity. Indeed, only a non-statistically significant trend was observed in terms of the occurrence of higher CV diseases and malignancies. Similarly, post-LT NASH patients’ data are discordant. However, in the general population, MASLD undoubtedly influences mortality and morbidity, and post-LT metabolic alteration has been associated with higher CV morbidity and fibrosis progression [41,67,179,180]. These discrepancies may be due to some causes: a too-short post-LT follow-up period (indeed, only a few studies include a follow-up of greater than 10 years), a potential underestimation of both the risk and the impact of steatotic disease when not encompassed within the frame of MASLD, substantial heterogeneity among studies in terms of diagnostic tools, protocol versus non-protocol assessments, and inhomogeneous cohorts with different proportions of baseline liver diseases.

Third, in the post-LT setting, MASLD may present a different natural history compared to pre-LT MASLD or post-LT NAFLD, as some studies have partly suggested. Dumoriter et al. reported a high incidence of fibrosis, even in the absence of inflammation, a rare occurrence among the general population [70]. Moreover, in some NAFLD/NASH patients, the development of fibrosis has been described as being faster than in non-LT patients, with significant fibrosis occurring even within the first year after transplant [32,35].

Concerning the role of risk factors, it must be reiterated that the importance of their detection lies in the intrinsic behavior of liver disease, which remains clinically silent until progression to advanced stages and decompensation. Therefore, the early identification of risk factors is essential for implementing preventive measures.

Since several studies have shown that the genetic background of both donors and recipients can play a role in the development of steatotic disease, a more standardized evaluation of genetic aspects before LT should be warranted. Indeed, this might lead to ideal genetic matching between donor and recipient to reduce the risk of MASLD occurrence.

Donor factors are marginal in post-LT NAFLD occurrence, while a younger age of the donor, transplantation from living donors, graft steatosis, and the donor’s BMI have been associated with post-LT NASH [12,26]. Regarding recipient factors, recurrent disease, steatohepatitis, a younger age, and the female sex have been associated, in different grades, with a higher occurrence and risk of progression of post-LT NAFLD [12,17,21,25,30]. Pre- and post-LT metabolic factors (primarily body weight) strongly predict post-LT NAFLD occurrence. This suggests that recipient features may have a greater impact than donor features, as proposed by Boga et al. [181]. In the frame of MASLD, confirming these risk factors or identifying others, such as sarcopenia—which has progressively gained interest in the past years—would be fundamental to recognizing high-risk patients and thus planning a more intensive management [66,182].

Immunosuppressants play a crucial role in the LT setting; therefore, tailoring and minimizing these regimens are paramount for improving long-term graft and patient survival. Their heavy impact on metabolic homeostasis is widely recognized, but unexpectedly, no significant association between IS therapy and steatosis development has been clearly identified so far. Indeed, this could be due to the presence of confounders or selection bias. More studies to elucidate this specific issue are needed. Meanwhile, rapid steroid tapering and lowering the dose of CNIs, alone or in combination with other immunosuppressants, or their switch are safe and effective interventions to mitigate metabolic alterations [28].

NITs have globally become increasingly useful to assess liver diseases. In non-transplant settings, most of them have been assessed and validated, with excellent performance [88,183]. Although liver biopsy remains fundamental in the LT field, the validation of NITs for assessing both fat accumulation and fibrosis stages may reduce the health-care-related burden in the LT setting, saving time, improving patients’ compliance, and reducing medical costs. In this regard, MR techniques seem the most accurate but with the limits of high cost, time-consuming examination, and patient acceptability. For these limitations, the most promising NITs are those based on US techniques, such as VCTE with CAP and the recent SWE, given the low cost, wide availability, high accuracy, and reproducibility.

Serum biomarker data in the LT setting are scarce, and generalizability from the pre-LT field is bound to be highly inaccurate. Unfortunately, validation studies of these NITs have not been performed, and this gap should be closed soon, particularly for steatosis assessment, whose definition appears less solid than that of fibrosis evaluation.

Concerning management, multidisciplinary teams are essential in the setting of LT due to the extreme complexity of the picture. Preventive measures should be the core of every intervention, mostly based on patients’ lifestyle modifications and education. Because of interconnected pathophysiological mechanisms, MASLD/MASH coexists with metabolic alterations and CV diseases. For this reason, an ideal therapy should not only improve liver disease with liver fat, inflammation, and fibrosis reduction but also improve the underlying metabolic state, ultimately reducing body weight and CV risk factors. In this sense, GLP1RAs alone or in combination with GIP agonists may be considered a backbone of the therapy soon, since these drugs, beyond improving liver histology, may reduce both body weight and CV risk factors. Prospective studies in the LT setting are urgently needed to confirm improvements in liver steatosis and fibrosis, as well as the hardest endpoints of both liver and non-liver outcomes, coupled with long-term tolerability and safety. In fact, the reported gastrointestinal events, i.e., vomiting, nausea, and diarrhea, may interfere with immunosuppression levels, while there is a lack of evidence from meta-analyses regarding the increased risk of acute pancreatitis and various forms of cancers.

Other anti-fibrotic and anti-inflammatory drugs may become pivotal in the LT setting, as well as other treatment strategies, such as gut microbiota manipulation and bariatric interventions, which are in rapid growth and probably will occupy an even greater clinical space [184,185].

Finally, another fundamental point concerns awareness of the disease. Indeed, although steatotic liver disease is not only highly prevalent but also progressively increasing, awareness is generally low among non-liver health specialists and particularly among patients. Although important improvements have occurred in the past years, other major steps are essential since awareness is the prerequisite for the global, effective management of any condition.

## 7. Conclusions

The development of recurrent and de novo steatosis after LT is a frequent condition that needs to be assessed through the more comprehensive scenario of MASLD. Indeed, hepatic fat accumulation and metabolic alterations often occur together and share the same pathogenetic pathways. A considerable fraction of these patients develop more severe forms of the disease that lead to steatohepatitis and fibrosis, which may progress to cirrhosis in a marginal portion. Although in these patients, the presence of steatosis/steatohepatitis does not appear to impact the overall survival heavily, there is scattered evidence of higher morbidity in terms of CV disease and malignancies’ occurrence. Therefore, the early identification of risk factors and prompt diagnosis are imperative for effective management. Some NITs offer reliable, easy-to-use, and repeatable options for monitoring liver transplant recipients, along with histological evaluation, which remains the gold standard. Management strategies rest on lifestyle modifications, the backbone of both preventive and curative interventions, but surely, rigorous control of metabolic alterations, first of all weight gain and obesity, by pharmacotherapy and the optimization of immunosuppressive regimens remain pivotal in minimizing metabolic derangements and mitigating the risk related to CV events, malignancies, and graft function.

In conclusion, an integrated, multidisciplinary approach is the base for the correct and effective management of MASLD in LT recipients, incorporating advanced NITs, targeted therapies, and comprehensive patient education.

## 8. Future Directions

As the burden of MASLD will probably continue to increase in the future, following the trend of the past decades, larger, prospective, and long-term studies with definite inclusion and diagnosis criteria are warranted to better characterize the incidence and economic burden of recurrent and de novo MAFLD and MASH. A better understanding of pathophysiological mechanisms and risk factors should be encouraged as they are the core of present and future management strategies. Improving NITs is essential in LT settings; indeed, reliable, easy-to-use, and repeatable diagnostic tools are more efficient in terms of patient tolerability, the use of healthcare facilities, and economic sustainability. About management, enhancing preventive strategies will be crucial to reducing the health burden. In this regard, greater awareness at the population level is desirable, and hospital or institutional campaigns could help. The effort of future studies should also converge on developing safe treatment in the settings of LT, too. In the frame of tailored medicine, creating standardized pathways that combine preventive interventions, specific medications, and interdisciplinary counseling will be essential to improving compliance and treatment efficiency. Additionally, more studies on invasive and minimally invasive interventions, such as surgery and bariatric endoscopy, are desirable in this field to determine which are the safest and best options.

## Figures and Tables

**Table 2:** Metabolic impact of the immunosuppressive classes used in LT.

	Corticosteroid	CNIs (Tac, CyA)	mTORis (EVR, SIR)	Antiproliferative Agents (MPA, AZA)	Monoclonal Antibodies (Basiliximab, Thymoglobulin)
Main physiopathological mechanism	↑ IR ↓ Insulin secretion ↑ Lipogenesis ↑ Gluconeogenesis ↑ Appetite	↓ Insulin secretion Altered lipid metabolism ↑ Vasoconstriction	↑ IR Altered lipid metabolism	Minimal direct metabolism effect	-
Weight gain	+	+	-	-	-
Hyperglycemia	+	+ (>Tac)	+	-	-
Dyslipidemia	+	+ (>CyA)	+	-	-
Arterial hypertension	+	+	+	-	-

↑: Increase, ↓: decrease. Abbreviations: insulin resistance (IR); calcineurin inhibitors (CNIs); tacrolimus (Tac); cyclosporine (CsA); mycophenolic acid (MPA); azathioprine (AZA); mammalian target of rapamycin inhibitors (mTORis); everolimus (EVR); sirolimus (SIR).

## Data Availability

Data supporting reported results are available among included studies. Individual patient data may be available upon request and agreement with the original study’s authors.
